# Understanding Physiological and Degenerative Natural Vision Mechanisms to Define Contrast and Contour Operators

**DOI:** 10.1371/journal.pone.0006010

**Published:** 2009-06-23

**Authors:** Jacques Demongeot, Yannick Fouquet, Muhammad Tayyab, Nicolas Vuillerme

**Affiliations:** TIMC-IMAG, UMR UJF/CNRS 5525, University J. Fourier of Grenoble, La Tronche, France; University of Southern California, United States of America

## Abstract

**Background:**

Dynamical systems like neural networks based on lateral inhibition have a large field of applications in image processing, robotics and morphogenesis modeling. In this paper, we will propose some examples of dynamical flows used in image contrasting and contouring.

**Methodology:**

First we present the physiological basis of the retina function by showing the role of the lateral inhibition in the optical illusions and pathologic processes generation. Then, based on these biological considerations about the real vision mechanisms, we study an enhancement method for contrasting medical images, using either a discrete neural network approach, or its continuous version, i.e. a non-isotropic diffusion reaction partial differential system. Following this, we introduce other continuous operators based on similar biomimetic approaches: a chemotactic contrasting method, a viability contouring algorithm and an attentional focus operator. Then, we introduce the new notion of mixed potential Hamiltonian flows; we compare it with the watershed method and we use it for contouring.

**Conclusions:**

We conclude by showing the utility of these biomimetic methods with some examples of application in medical imaging and computed assisted surgery.

## Introduction

*“In nova fert animus mutatas dicere formas corpora…” I want to speak about bodies changed into new forms… (Ovid, Metamorphoses, Book 1^st^, 10 AD).*

**Figure 1:**
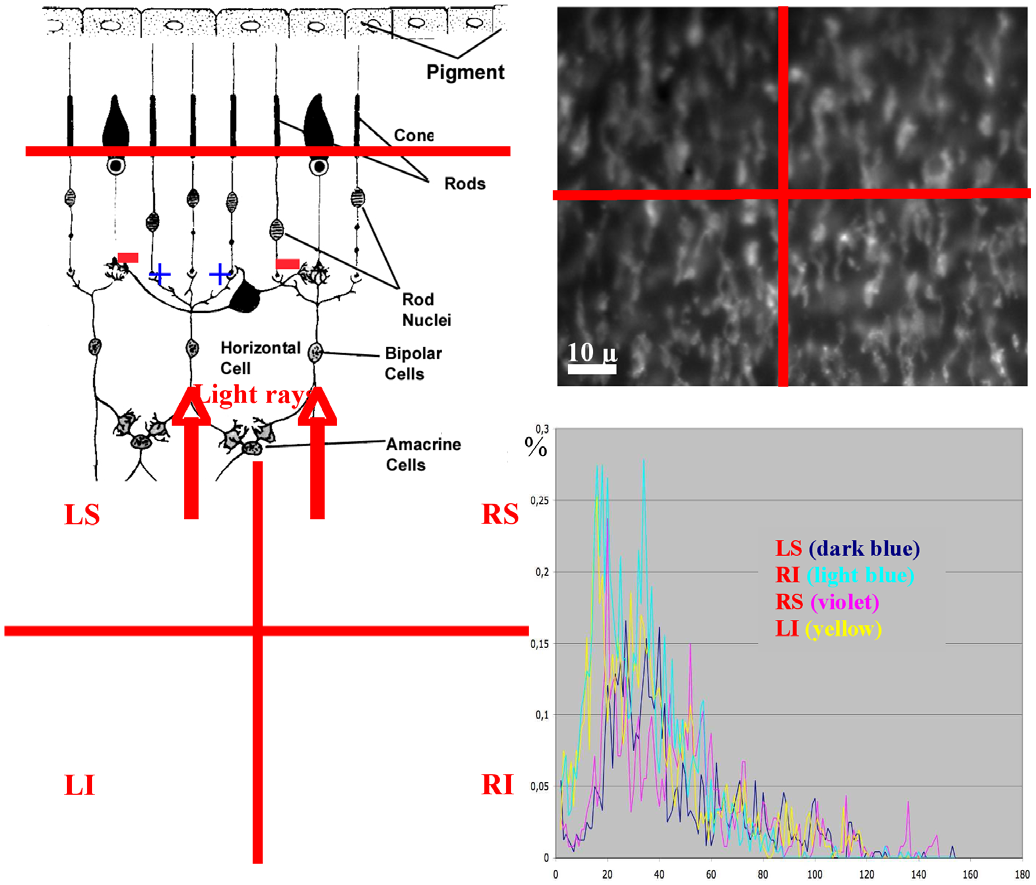
Physiological and pathological retina. Top left: lateral inhibition due to horizontal cell synapses [after 3]. Top right: confocal microscopy slice of mouse retina with retinitis pigmentosa coming from T. Léveillard & J.A. Sahel [19]. Bottom left: segmentation of cones and rods with a cell deficit in the quadrant Left Superior (LS) [34]. Bottom right: histogram of the intercept distances showing an augmentation of the inter-cell distance in the quadrant Left Superior with respect to others Left Inferior (LI), Right Superior and Inferior (RS & RI) [34].

**Figure 2:**
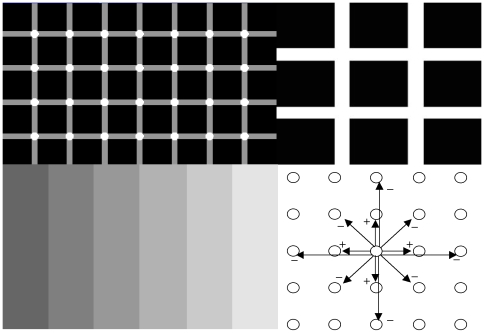
Contrast illusions. Top left: Hermann illusion with bright points at the intersection of grey stripes. Top right: Hermann illusion with grey squares at the intersection of white stripes. Bottom left: Mach bands illusion with enhancement of the vertical lines separating the different grey zones. Bottom right: lateral inhibition with activation at short range (nearest neighbour neurons) and inhibition at medium range (second Manhattan sphere)

**Figure 3 pone-0006010-g003:**
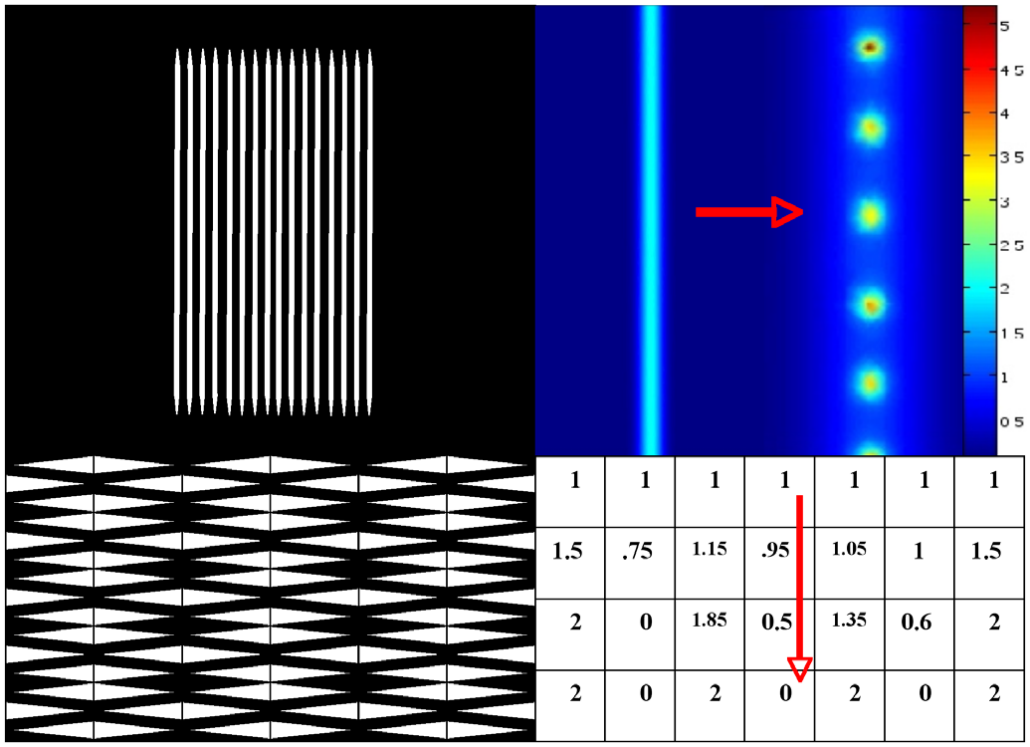
Contrast illusions. Top left: illusion of bright reinforcement at extremities and middle of white stripes in tangential vision. Top right: progressive change of a vertical bright stripe into bright spots (in false colours) during a morphogenetic process with lateral inhibition of morphogens [91]. Bottom left: bright and grey activities, respectively, near the center (vertical black line) and the extremities of the white horizontal diamonds. Bottom right: lateral inhibition simulated by a simple threshold neural network with θ = w_ii_ = 2, w_ii−1_ = w_ii+1_ = −0.5 and a sequential updating from the left to the right hand side

**Figure 4:**
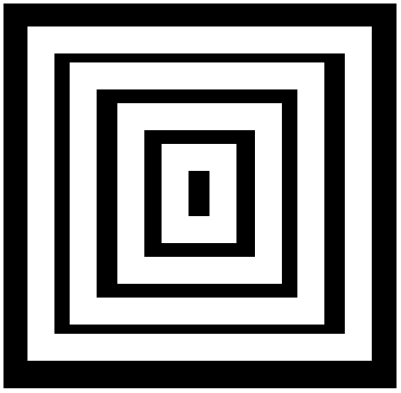
Contrast illusions. Kanizsa pyramid: the lateral inhibition causes the sensation of seeing a 3-dimensional pyramid.

In the vertebrate retina, cones are hyperpolarized when illuminated by light, but also receive a depolarizing input when receptors some distance away are illuminated. This antagonistic center-surround response is mediated by amacrine and horizontal cells (Figure 1), through a sign-reversing synapse to the cones often called feedback synapse, the global mechanism being called lateral inhibition [1]–[3]. This surround response is involved in edge enhancement and image contrasting [4]–[16] realizing concretely the Mach (boundary brightness overshoot) and the Marr (Laplacian zero-crossing edge-enhancement) effects, used in many image processing applications [17]. A number of contrast illusions (Figures 2, 3, 4) have been described [18] based on the lateral inhibition principle. In order to examine how rod and cone functions are differentially affected during retinal degeneration (abolishing the contrast), many studies have been done on the genetic level showing that these two cell types have complementary roles during both development and degenerative processes [19]–[21]. For understanding the retinal physiology as well as this pathology, many models [22]–[34] are now available which try to mimic relevant adaptation behaviours of the human visual system, like lightness/colour constancy and contrast enhancement, corresponding to the ability of the visual system to increase the appearance of large-scale light-dark or inter-colour transitions, similar to how sharpening with an “un-sharp mask” increases the appearance of small-scale edges.

These models use theoretical developments [35]–[44] in dynamical systems, especially the study of their attractors. An attractor represents the ultimate evolution of a dynamical system when time tends to infinity; after perturbations, an attractor recovers its stable dynamical features, like its period and amplitude. That requires a rigorous mathematical framework for defining the continuous flow and its convergence speed to attractors, and after its discrete version, i.e. an iteration process representing the succession of states of the dynamical system. These theoretical advances have permitted the development of fast image processing algorithms used in rapid contrasting methods [45]–[58] implemented in real-time processors [59]–[68], and the development of contouring methods like snakes, snake-splines, δ-snakes, which allow a global definition of the boundaries of objects of interest in an image. These algorithms have emphasized the role played by computer implemented procedures, starting from an initial compact, e.g. a sphere, and ending at the final shape of the object's contours after a certain number of iterations [69]–[80]. The corresponding flow is a compact set valued flow, the simplest deriving from a potential [81]–[86]. In general, this methodology allows one to rapidly and automatically obtain 3D contours, which is necessary in medical imaging to perform computer aided medical interventions. If the dynamics are conservative in a neighbourhood of an attractor, the flow becomes Hamiltonian, so we then will define the notion of mixed potential Hamiltonian flow. This flow gives a theoretical support to the Waddington's notion of chreod, particularly relevant in embryonic morphogenesis modeling [87]–[91], but also serves in image contouring.

Using the previously introduced theoretical notions, we study an enhancement method for contrasting medical images, using either a discrete neural network approach, or its continuous version, i.e. a reaction-diffusion partial differential system [92]–[99]. Indeed, having the goal of providing for a rapid and efficient action [100]–[142] in precise surgical robotics as well as in disease diagnosis and satellite control imaging, such pre-treatments are performed for contrasting and then contouring images. The medical community, for example, often uses pre-treated anatomical images coming from imaging devices, like MRI or CT-scanner, whose pre- processing involves two fundamental steps: contrasting and contouring. The natural vision executes these two tasks, the first one being based on the architecture of the retina, which uses lateral inhibition to reinforce the perception of the contours of homogeneous objects in a scene. Because the objects of medical interest are homogeneous with respect to their environment (a tumour or an organ are made of cells coming from the same cellular clone), they are well enhanced by using operators processing as in the natural vision. Therefore, we introduce continuous operators generalizing discrete neuromimetic approaches using lateral inhibition as well as analogs of the Hebbian rule for the evolution of synaptic weights.

## Results

The results presented in this Section involve consecutive phases of contrasting and segmenting in order to identify objects of interest in an image. The important features of a scene are the prey, predators, and sexual partners. For the detection of these features, the major characteristics are the “phaneres”, this word coming from the Greek phaneros: visible. The “phaneres” in animals and plants are prominent visible tegumentary formations like feathers, scales, hair, petals, skin spots and stripes of various forms and colours. The role of the contrasting pre-treatment in the retina is to rapidly enhance the characteristics (luminance, colour and texture) on the boundaries of the homogeneous zones in a scene in order to improve their perception and extract the features associated to the vital functions like the nutrition, the survival and the reproduction. This process can trigger very fast actions (like escaping a predator) after a stimulus of about 150 ms [136]. Such fast sensory-motor loops need a very simple and rapid mechanism well encoded in the anatomy and in the physiology of the retina (like the center-surround response of cones and rods [1]–[3]), early before a semantic recognition and denomination of the prey or of the predator. We will give first some results concerning the natural contrasting process both in a natural and in a simulation context.

### Pathologic retina

The lateral inhibition mechanism in the retina is due to the presence of feedback synapses of horizontal cells [1], [2], which reverse the sign from activation of the cells surrounding that were illuminated (Figure 1 top left). The retina pathologies provoke a progressive death of rods (as in retinitis pigmentosa) followed by the apoptosis of the cones; then, the non-secretion by rods of a growth factor favouring the cones survival, causes the disappearance of the lateral inhibition, hence of the contrasting ability [4], [19], [20], [21]. As shown in the top right and bottom left of Figure 1 on a confocal slice of a sick retina, we observe an important loss of both rods and cones in the left superior quadrant. An analysis of interdistances among cells in the three other quadrants shows that the mean interdistance between cones in the peripheral retina (about 20 µ) is better conserved than the corresponding value between rods (about 3 µ), proving the primary rod degeneracy.

### Contrast illusions

The perception of artefactual stripes or spots comes from the lateral inhibition effect, which causes a reinforcement (respectively decline) of brightness in a pixel if its neighbours are black (respectively white). This illusion effect is visible on the Figures 2 to [Fig pone-0006010-g003] 4. In Figure 2 (top-left), the Hermann illusion is provoked by the local organization of inhibition and activation between retinal cells, which is described bottom right. The illusion shows bright squares at the intersection of grey stripes and grey squares at the intersection of white stripes. In Figure 2 (bottom-left), the Mach bands illusion gives an enhancement of the vertical lines separating the different grey zones. In Figure 3 top-left, the tangential vision (which allows to escape the macular vision) gives the illusion of a bright reinforcement at the extremities and middle of the white stripes. On the top right, a progressive change of the vertical bright stripe into bright spots (in false colours) is observed during the feathers morphogenetic process in chicken due to a lateral inhibition effect between morphogens (model and simulation are given in [91]). On the bottom left, we can observe bright and grey activities respectively near the center (vertical black line) and the extremities of the white horizontal diamonds. For explaining these illusions, we can simulate a very simple threshold formal neural network (cf. infra) made of 7 neurons, with a lateral inhibition mechanism defined by the parameter values θ = w_ii_ = 2, w_ii−1_ = w_ii+1_ = −0.5, and a sequential updating from the left to the right hand side. The spots activity appears after 3 iterations as a stable steady configuration, and is the discrete analog that the feature created by simulating the continuous reaction-diffusion operator used for modelling feathers morphogenesis [91]. In Figure 4, the sensation of seeing a 3D pyramid is the generalization of the well known Kanizsa polygon effect. It is due to the artefactual prolongation of the white square extremities as white (respectively black) lines in a black (respectively white) dominant neighbourhood. The illusion effects described above are easy to simulate by computer and can serve as external efficacy criterion when different contrasting methods are benchmarked.

### Contrasting and contouring images

**Figure 5:**
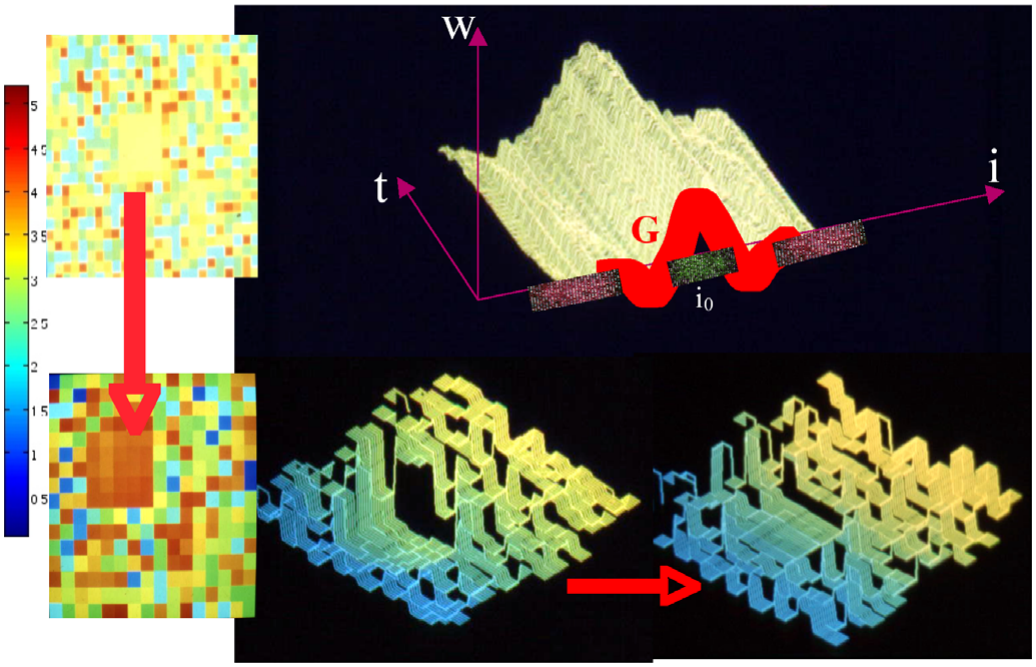
Contrast enhancement. Left: the lateral inhibition causes the enhancement of the yellow square (with medium level in false colours) in a contrasted bright orange square [48]. Top right: temporal evolution of the Difference of Gaussian function representing an activation near the central neuron i_0_ (green links) and an inhibition (red links) farther from i_0_ [47]. Bottom right: same processing in grey level with initial image on the left and contrasted on the right [49]

The enhancement of the grey level on its maximal gradient lines (identical to the geometric locus formed by all the points where the mean Gaussian curvature on the grey surface vanishes) is due to the retinal processing and causes the sensation of contours. By using an enhancement procedure based on the lateral inhibition effect in an formal neural network receiving as input the grey level of an image, we have obtained a good contrast on the boundaries of homogeneous zones either on simulated or on real images. Figure 5 (respectively 6) shows the result obtained after applying a contrasting algorithm on an artificial image (respectively on the NMR slice of a brain tumour).

**Figure 6:**
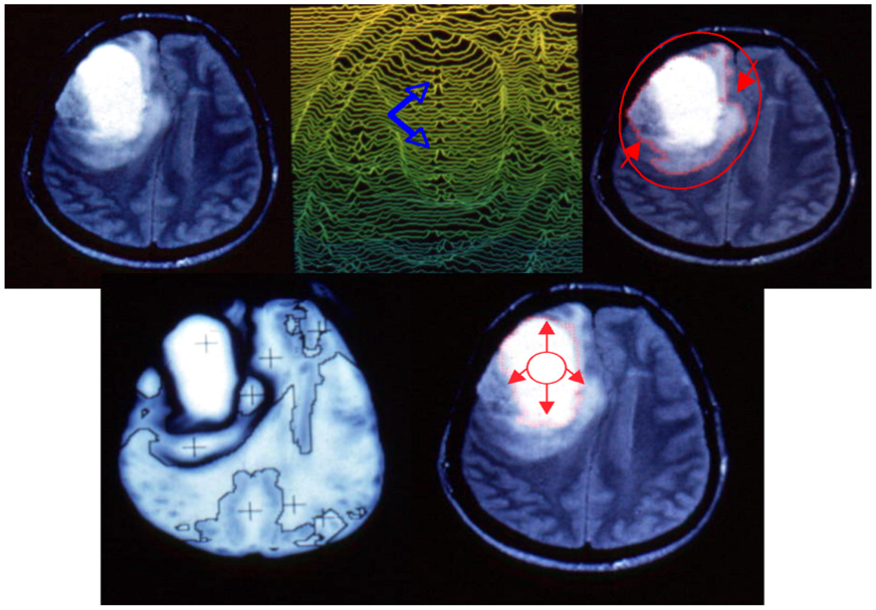
Contrasting and contouring medical images. Top left: initial NMR image of a brain tumour [48], [49]. Top middle: contrast enhancement with occurrence of a central activity (blue arrows). Top right: boundary of the compressed tissue (using a snake spline). Bottom left: tumour segmentation. Bottom right: tumour boundary (internal snake spline)

**Figure 7:**
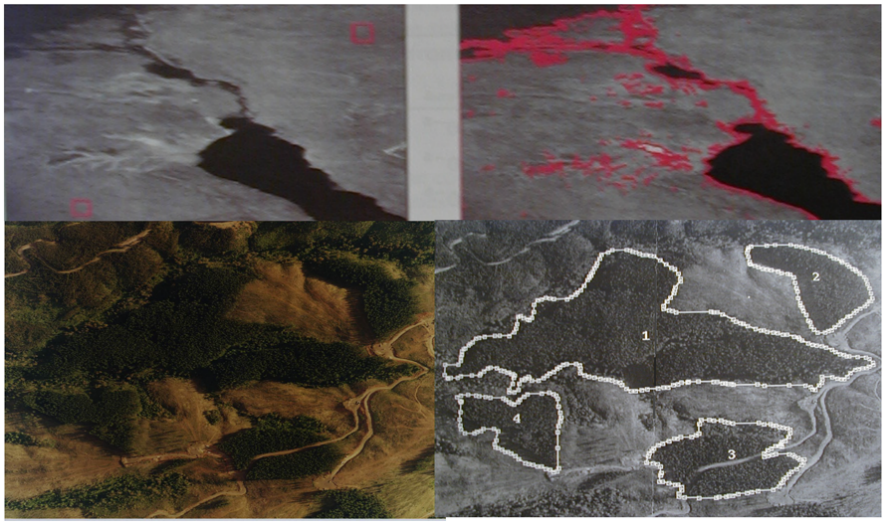
Contrasting and contouring satellite images. Top left: compact flow initialization [83]. Top right: contour of Suez Canal [83]. Bottom left: image of Chilean forest. Bottom right: contrasted image using chemotactic operator and snake-spline contouring [85], [142]

The contouring step follows the contrasting one, and we see in Figures 5, 6 and 7 contours of homogeneous (in grey level) zones resulting from a snake-spline procedure (i.e. an external snake-based procedure with the constraint to keep a spline closed curve at each step) applied over an artificial isolevel square (Figure 5), a brain tumour (Figure 6) and a forest (Figure 7) made of the same species of elements (pixels, cells and trees respectively).

The two steps of contrasting and contouring are based on classical algorithms of neural networks [24], [31], [32] and snake spline [69]–[76], but they can involve new methods coming from biomimetic procedures. We will describe rapidly four such new methodologies and give examples of their application to real satellite or medical images.

**Figure 8:**
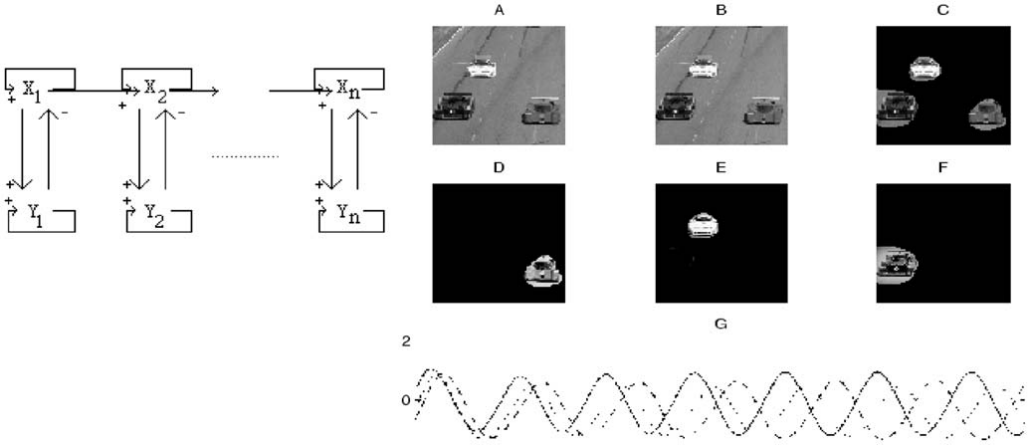
Image attention processing. Top left: hippocampus-like neural network with lateral mixed action. Top-right: from A to F, progressive attentional focus by locally synchronizing the periodic signal associated to each pixel [56]. Bottom-right: desynchronization process between periodic activities of the neurons X_i_ (i from 1 to n)

1. **A chemotactic operator.** If we denote, at time t and pixel x, g(x,t) as the grey level function, we can consider g as a food or substrate, which living entities (like bacteria) can eat, being attracted from the image boundaries (where they are first located) by a chemical gradient linked to the substrate. Let us denote the bacterial concentration by b(x,t). We can consider the following equations, which constitute a new image processing operator [85], [142]: 
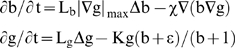
(1)with Neumann conditions on the image boundary, where 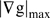 denotes the maximum value of the g gradient norm, χ is the attractive chemotactic constant, L_b_ (respectively L_g_) is the diffusion coefficient of the bacterial concentration (respectively grey level), K (respectively Kε) is the maximal (respectively minimal) grey consumption rate of bacteria. These equations imply that the bacteria move towards the concentration of grey considered as a chemo-attractant to consume. They also diffuse as the grey level with respectively the diffusion constants L_b_ and L_g_. The Figure 7 bottom shows the progressive treatment of the image of a Chilean forest presenting the same characteristics of internal homogeneity as a tumour (the trees replacing the cells); due to the fact that the trees (like cells) belong to the same genetic lineage. After reaching their asymptotic values, the dynamics of contrasting implemented in a discrete scheme of the partial differential equations (PDE) (1), stops and this processing step can be followed by a snake spline contouring step.
2. **A viability contouring operator.** If we minimize the following function, 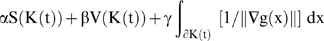
(2)we obtain a new snake operator [75], [85], where K(t) is a compact object of interest moving toward a limit set K(∞), whose external surface S as well as its inner volume V are minimized, allowing a contouring with real gloves (precise contour) contrarily to mittens (convex envelop) often observed with the Mumford-Kass-Terzopoulos algorithm in Figure 7 [69], [70]. We see in Figure 7 (top and bottom right) the contouring done by imposing a bicubic spline to the boundary at each time step [71], [72], followed by a 3D spline smoothing. Many other approaches can also be used for controlling the active-shape models. This is the case in the level set methods used for computing and analyzing the motion of an interface in two or three dimensions by modelling the velocity vector field through Euler-Lagrange or Hamilton-Jacobi PDE's [77], [78], [79], [80]. These PDE's can be used to model the segmentation of a moving 3D object (like the heart) giving a particular status to the pixels having a maximal velocity or acceleration of their grey levels. This procedure has been used for segmenting the pericardium [131].
3. **A non-isotropic reaction-diffusion operator.** If we consider the grey level function g(x,0) as the initial image, we can follow the transient behaviour of the non-linear diffusion operator defined in [93]: 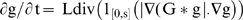
(3) Here G is a Gaussian kernel of fixed variance and with Neumann conditions. Its asymptotes correspond to a constant grey level suppressing the objects of interest inside the image. For that reason, we consider now a non-isotropic reaction-diffusion operator defined in [93], [95], [96]: 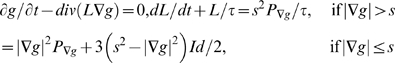
(4) where L is a 2×2 matrix and P_∇g_ is the orthogonal projection matrix: 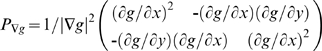
(5) In the equations above, the diffusion constant L becomes variable with the time t and its evolution equation is similar to the Hebbian rule of a discrete neural network operator. Treated images are obtained at the asymptotic state of the PDE dynamics as for neural networks [48], [49] with lateral inhibition (Figure 6). A comparison done in [96] shows that the asymptotes of this non-isotropic operator are better than for some of the operators described earlier. More generally, we can notice in the other PDE approaches:
4.
  - The application of the pure heat operator [145] quickly leads to a constant grey level
  - In the Perona-Malik operator [92], the viscosity is different within a region and across its boundary in order to encourage smoothing inside the region of interest; this operator can be used transiently for this purpose before the non-isotropic reaction-diffusion operator
  - The Catté-Lions-Morel-Coll algorithm [94] gives a good contrasting during the transient behaviour of the operator, but has the same asymptotes as for the pure heat algorithm (even it is reached more slowly)
  - The non-isotropic reaction-diffusion operator [93], [95], [96] offers a reasonable asymptotic processing
  - The Weickert operator [97] permits the completion of interrupted lines or the enhancement of flow-like structures by choosing the appropriate smoothing direction in anisotropic processes in spirit to the Cottet–Germain filter [95]
  - The Tschumperlé-Deriche operator [98], [99] allows the regularization of velocity vectors fields in 4D imaging (acquired for example during the motion of a 3D camera).
5. **An attentional focus operator.** For focusing on only one region of interest, we have to change the image input on an artificial neural network [56]. This input can be constant [24], [31], [32], stochastic [47]–[54] or deterministic periodic [56]. This last coding mimics the information storage inside the hippocampus in which the functional unit, made of two neurons in mixed inhibition/activation interaction (Figure 8 top left) has an attractor limit cycle. We can locally synchronize, using an evocation stimulus, and desynchronize, by introducing noise on the inter-unit interactions, the periodic activities corresponding to initially non phase-locked neurons. In this way, we enhance considerably (by forcing the units to add their maximal activities at the same time) the grey level on the zones of local synchronization (Figure 8 E bottom right). Then, by thresholding and segmenting, we get the parts of the initial image (Figure 8 A top right) on which the attentional focus has been exerted (Figure 8D, E, F top right).

### Computer assisted interventions

**Figure 9:**
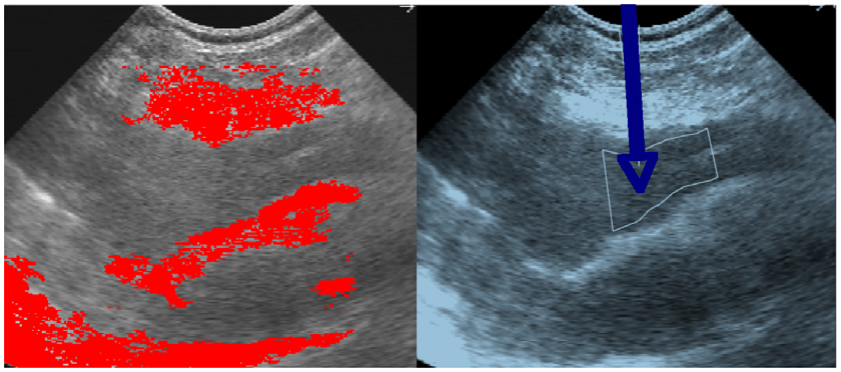
Computer assisted interventions. Left: Use of the confinement tree for delimitating security regions (red) in an ultra-sound image before computer assisted puncture [131], [132]. Right: zone chosen for introducing an external needle for puncturing a pericardial effusion [132]

For introducing and driving medical or surgical tools (like needles, electrodes, bistouries) into the human body [118]–[135], one needs to segment and contour (after contrasting) zones of interest to avoid (as indicated by red zones in Figure 9 left representing tissues of lungs on the top and cardiac muscle on the middle and bottom) or to reach (blue arrow in Figure 9 right indicating a pericardial effusion). This example gives a good illustration of what can be exploited from the contrasting and contouring operators in order to go farther than the descriptive level for diagnosis. That is to really improve some medical procedures, one must automate the process completely, thus replacing the human actor without any loss of speed or precision [119]–[130].

## Discussion

### Interest of the biomimetic approach

The biomimetic approach used in numerous methods presented in this paper, especially for the contrasting phase, exploits the efficiency of visual data processing procedures that have been selected by natural evolution. These procedures represent an optimum in terms of economy of implementation (small number of living elements involved, like cells, tissues, vessels, etc), speed and precision. They also are based on operations that come after processing by the retina and visual areas, thus providing high level semantic neural networks that define the symptomatology related to the observed medical reality. The extraction of semiotic characteristics of objects of medical interest that have been enhanced and contoured using biomimetic methods allows medical signs and symptoms to be organized in syndromes, thus facilitating the diagnosis process. The concept of biological information encoded in a genetic program that controls development forms a major part of the semiotic metaphor in biology. The development plan is seen as being analogous to a computing program, and “semiotics of nature” studies the structural relations as explored by molecular and evolutionary biology [137]. Y.L. Kergosien [138] advocates a semiotics of nature in an epistemological sense for analysing interacting biological systems, in order to increase the precision of terms such as “signal” in biology or “symptom” in medicine, and to develop new themes of inquiry into the nature of their biological or medical signification. The Kergosien approach indeed allows for a concept of natural signification. The adaptation of an animal to a specific function is seen as the realization of the natural metaphor [137]. This is the case for retinotopically arranged neuronal sets that code for homogeneity features (brightness, colour, texture, etc), oriented contours, and corners of an object. Simultaneous representation by colour neurons, complex model neurons (with oriented receptive fields), and hypercomplex model neurons (responding to corners) makes attention and recognition robust and reliable, in the framework of emergent abilities of optimized complex systems [139]–[141].

The bio-inspired image processing methods also have a tendency to use an information encoding that provides for optimum information storage and query, as done in mnemonic structures like hippocampus. In general these structures possess their own formats of information encoded in periodic temporal neuronal activities that we can mimic to optimize both compression and retrieving procedures [40]. All these neural treatments can induce illusions and artefacts. But the knowledge about their origin can be used for preventing such abnormalities in the low level (contrasting and contouring) as well as in the high level (semantic assignation and recognition) image processing steps. The neural treatments need also to avoid pathologic processing, due to a non-optimal number of their neurons and/or interactions and to a non robust value of their parameters. To that precise purpose, a deep scientific knowledge about the physiology and the pathology of the retina constitutes an unavoidable inheritance.

### Limits of the biomimetic approach

In order to be faster, the methods mimicking the natural process of vision need to be made parallel as in the real neuronal systems. But the attractors of the dynamical systems permitting contrasting and contouring of the images are highly dependent on their modality of implementation, particularly on their updating mode. In general, the fixed configurations obtained by simulating such systems are robust with respect to the mode of updating, but it is not the case for the periodic neural activity we have used in attentional focusing (Figure 8). Hence it is convenient to be very careful until the final step of algorithmic implementation.

The imitation of nature does not push to avoid theoretical studies on the spatio-temporal processes used in artificial vision [142]. Only this fundamental approach is able to finally guide the methodological choice with arguments as fast calculation speed [143], precision, accuracy, and minimal algorithmic complexity. Indeed, these good properties constitute the main criteria for selecting robust, fast and precise image processing tools for reliable procedures of computer aided surgical and medical intervention [118]–[135].

## Materials and Methods

### Discrete operators

#### Contrast enhancement

A large number of methods of contrast enhancement have been used in the past to reinforce the grey level gradient on the boundaries of objects of interest. These methodologies can be classified following a typology, based on the mathematical tools underlying algorithms:

- classical filtering (e.g. Gaussian [28]), PDE filtering [77], [78], [79] the simplest being the heat operator analog for the Gaussian filter, grey histogram thresholding [26], [30], entropy techniques [25], adaptive filtering [64]

- multi-scale, in particular wavelets [84]) approach [27], [31]

- fuzzy clustering [29]

- specific hardware implementation [60], [62] for real-time procedures [22], using either the simplest PC based [23] or the most sophisticated architectures (SIMD [32], [52] or MIMD [68])

- neural networks techniques both discrete [48]–[55] and continuous [93], [95], [96].

We will focus in the next Section on the neural networks techniques which are the closest to the natural vision processing.

#### Definition of a formal neural network

A formal deterministic neural network R of size n is defined by its state variables {x_i_(t)}_i = 1,_ …_,n_, where x_i_(t) denotes the state of the neuron i at time t (equal to 1 if the neuron fires at this time and to 0 if not). Then the discrete iterative system ruling the change of states in the network is given by the following equations: 
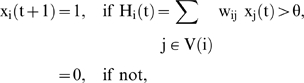
where V(i) is a neighbourhood of i, H_i_(t) plays the role of the somatic electric potential, w_ij_ designates the synaptic weight representing the influence of the neurone j on the neurone i and θ is a firing threshold. The updating of the neuronal states can be operated:

- either sequentially, after having chosen a certain order for the neurones,

- or block-sequentially, by operating the updating in parallel in each sub-network of a partition of R and by afterwards activating these sub-networks sequentially,

- or in a massively parallel fashion if only one sub-network exists.

### Input in a neural network

If an input I_i_(t) is sent to neuron i at time t, it is merged with the information coming from the neighbourhood V(i) in order to build the somatic potential H_i_(t): 
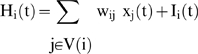



A very simple way of generating such inputs is to choose, for each time interval E_k_ (supposed to be independent of the others) between the two consecutive inputs 1, the k^th^ and the (k+1)^th^, the truncated geometric distribution: Prob({E_k_≤T_i_}) = 0 and Prob({E_k_ = m > T_i_}) = p_i_(1−p_i_)^m−Ti−1^, where T_i_ and p_i_ denote respectively the refractory period and the spike occurrence frequency on the afferent fiber i bringing the electric input to the neuron i. The truncated geometric processes are independent or correlated between fibers. In Figure 5, we can see the activity of a formal neural network activated by a non-homogeneous input representing the initial image (top-left), and after iterating the neuronal firing, we obtain as mean asymptotic behaviour (bottom-left). The coding is obtained by taking T_i_ and p_i_ proportional to the grey level of the initial image. Image on the top-right is representing the dynamics of the synaptic weights {w_i0j_(t)}_j∈V(i)_ which follows a Hebbian rule reinforcing the weight w_i0j_(t) if i_0_ and j had the same firing activity at time t: 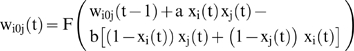
where F is a sigmoidal function of arc-tangent type. The initial distribution {w_i0j_(0)}_j∈V(i)_ is chosen dog-like (i.e. a difference of Gaussian distribution centred at i_0_, the negative Gaussian having the greatest variance as shown in the red dog G in Figure 5 (top-right)), for mimicking the lateral inhibition. The image treated is shown in grey level in Figure 5 (bottom-right), from initial to treated asymptotic image. We see that the square having a medial activity is enhanced by the lateral inhibition expressed by the dog function and its final level after iterating the network until it reaches its asymptotic firing regime, has a level clearly augmented (see the orange square on the bottom left and the enhanced “mesa” on the bottom right). Such a simulation highly suggests that an analogy between pixels and neurons can be made allowing the transfer of neural filtering techniques in image processing [24], [31], [48].

### Gradient enhancement by a neural network

#### Image enhancement procedure

We now present in 4 steps, the essentials of a method, easy to parallelize based on the same principles as proposed in [31]:

1. reduction of a 512×512 NMR image in a 256×256 image by averaging each block of 4 neighbour pixels, in order to obtain the input image (cf. Figure 6 top left).
2. use of this image as the mean configuration of an input geometric random field transformed by a 256×256 uni-layer neural network implemented in parallel; this network has an internal evolution rule, realizing a treatment of the input signal very close to a cardinal sine convolution, mimicking the lateral inhibition and favouring the occurrence of a very steep gradient on the boundary of homogeneous (in grey level) objects of interest in the processed image. In Figure 6, the object of interest is a brain tumour, its homogeneity coming from the same clonal origin of all its tumour cells.
3. use of the gradient, built by the neural network as the potential part of a mixed potential Hamiltonian differential system, whose Hamiltonian part is given by the initial grey level (before the action of the neural network).
4. obtaining boundaries of homogeneous objects as limit cycles of the differential system by simulating trajectories of the system in the different attraction basins.

The step 2 consists of defining the input from a geometric random field, i.e. a collection of geometric random processes such that, if p_i_(t) denotes the probability to generate a spike on the afferent fiber i to the neuron i at time t, we have: p_i_(t) = 0, if t−s_i_≤T_i_, where s_i_ is the time of the last 1 on the fiber i before time t and T_i_ denotes the refractory period, chosen as a constant equal to R. p_i_(t) = α_i_ sin_+_(ω_i_(t−s_i_−R)), if t−s_i_ >R, where sin_+_ denotes the positive part of the sine.

In order to incorporate an adaptation learning effect, a Hebbian evolution of the w_ij_'s is chosen based on the reinforcement of equal grey activities in the same neighbourhood: 
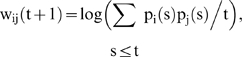
where w_ij_(0) values come from a dog (difference of Gaussian) distribution of j centred at i, for each i, for mimicking the lateral inhibition. This formula corresponds to the fact that w_ij_(t) is just the non-centred covariance function between the p_i_(s)'s and the p_j_(s)'s; if ω_i−_ω_j_ and R are small, w_ij_(t), when t tends to infinity, tends to log((α_i_α_j_/2)sin(ω_i−_ω_j_)/(ω_i−_ω_j_)).

### Image coding

After normalization of the grey level g(i) in the pixel i between 0 and 1, we take: 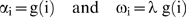
and we start the procedure by iterating the deterministic neural network. It is easy to prove that the probability π_i_ to have 1 as output of the neuron i at time t, just before renormalization, is about proportional to: 
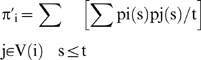



This last formula has been used to make the gradient enhancement visible in Figure 6 (top-middle). The behaviour of the function π'_i_ is similar to a convolution by a cardinal sine function, because of the approximate asymptotic formula: 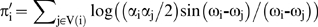



It is easy to verify that this convolution reinforces the “plateau” or “mesa” activities in grey level (or white if necessary). Such activities correspond, in medicine, to pathological objects to be considered as targets during the treatment (like tumour in which the same clone of cells gives a homogeneous response in absorbance or resonance) or to physiological objects (like a tissue made of cells having the same function) to be avoided during the treatment. Figure 6 shows the result of a gradient enhancement by the network for a brain tumour. Let us finally remark that we get objects treated at the asymptotes of the network dynamics. We do not need a stop criterion after few steps of processing and the method is easy to parallelize [55], [61].

### Continuous operators

The final aim of these methods is to offer a set of continuous operators adapted to segmentation of grey singularities or grey peaks (0-dimensional objects like micro-calcifications), grey anticlines (1-dimensional objects like vessels) or grey “mesas” (2-dimensional objects like tumours or functional regions). The problem of segmentation of more complicated objects (fractal objects like diffused tumours affecting, for example, the conjunctive tissue) is open and demands that other variables like texture based one's (e.g. the local fractal dimension or the wavelets coefficients) need be taken into account instead of or along with the grey level.

Let us consider now a compact state set E included in R^2^ and a temporal set T included in R_+_ or N, depending on the continuous or discrete version of time used. Let **K**(E) denotes the set of all compacts of E. If we provide **K**(E) with the Hausdorff topology (defined by the Hausdorff distance d between subsets), we can define **a compact set valued (csv) flow** Ø as a continuous application of **K**(E).T to **K**(E), which is a semi-group: 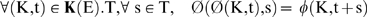



Because **K**(E) is a metric space, which is compact if E is compact, we can apply the operators limit and basin as defined in [36], [37] to the set valued flow Ø, and hence define the notions of attractor and of stability basin. We will give some examples of csv flows, whose attractors are objects to be contoured in image processing, or final shapes to be obtained at the end of any morphological development, these targets being often the same.

#### Potential flows

In snake contouring [69]–[72], the aim is to obtain the boundaries of an object of interest by progressively deforming the boundaries of an initial well-known set K(0) (e.g., a sphere) placed outside (respectively inside) the object, and whose deformation K(t) causes the decrease (respectively increase) of a potential function **P** [75] such as: 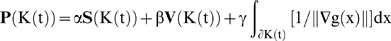 in which **S**(K(t)), **V**(K(t)), **∂**K(t), and g(x) denote respectively the external area, the inner volume, the boundary, and the grey level at the point x of the compact K for iteration t. The gradient iterations of **P** correspond to a discrete potential flow. For obtaining the continuous version, it suffices to use a potential “mutational” equation [81]–[83]. We can also add splines- like terms, e.g. δ∫**_∂_**_K(t)_C(x)dx, where C(x) = (∂^2^g/∂x_1_^2^)(∂^2^g/∂x_2_^2^ is the mean Gaussian curvature at x (in order to minimize the total variation of the local curvature like for the splines functions), plus a mean square criterion forcing **∂**K(t) to pass in the vicinity of points known *a priori* with fixed curvatures (in particular singular parabolic or saddle points, if their localization is known *a priori*).

#### Mixed potential Hamiltonian Segmentation

The continuous modelling allows stable evolution of differential operators such as gradient or Laplacian. Our segmentation consists in building a differential equation system whose stable manifold is the surface of the object we are looking for. Finding this manifold turns out to be a particular case of the surface intersection problem and provides an immediate analytical representation of the surface. The other major advantages of this method are to perform segmentation and surface tracking simultaneously, to describe complex structures in which branching problems can occur if the segmentation is purely local, and to provide accurate and reliable results.

Let us first consider the 2D problem. The central idea of the method is based on the Thom-Sebastiani conjecture [35] concerning the differential system: 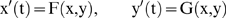



In the neighbourhood of a stable singularity or of a limit cycle of the corresponding velocity vector field supposed to be continuous, let us suppose that we can decompose the system into two parts, a potential and a Hamiltonian one, such as: 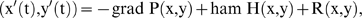
where the residue R(x,y) tends to 0 when (x,y) tends to the stable singularity or to the limit cycle. Such decomposition has been proven for a large class of Liénard systems [41]–[44]. The Thom-Sebastiani conjecture assumes that this result still holds by considering sufficiently regular systems. We will exploit systematically in the following, this possibility to consider a contour as the limit-cycle of a mixed potential Hamiltonian system. In fact, we consider now the boundary surrounding a 2D object with an approximately homogeneous grey level g, thus verifying: 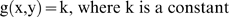



The corresponding curve is represented with parametric coordinates by:
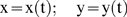



The continuous modelling implies the existence of the first derivatives of g; so a solution should verify the following equation obtained by differentiation of g(x,y)  = k:
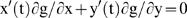



A particular solution of this equation is: x'(t)  = ∂g/∂y, y'(t)  = - ∂g/∂x, but this system does not provide a stable solution; a perturbation (due to noise) moving the curve away from the initial contour line could not be corrected. That is why we add a component which brings the curve back to the contour line defined by g(x,y)  = k, according to the steepest slope line of the function (g-k)^2^. We thus obtain:
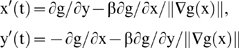



This system consists in two parts: the first one corresponds to an “edge tracking” component and the second one is a kind of “elastic force” which allows noisy image processing. The β parameter allows to balance these two terms. The system may be solved by numerical analysis methods with initial conditions, like the Runge-Kutta-Gear method. The parametric representation of the curve is then directly obtained. This continuous method can be applied in 3-dimensions to look for particular features of the surface of an object of interest. Let us consider such a surface defined by: f(x,y,g) = constant, parameterized by:
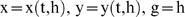



Our boundary tracking method can be implemented as follows: the algorithm starts with a point on the surface with a grey value h. For each slice of level h, the differential system is solved in order to obtain a closed curve. From some points of this curve, we follow the object surface until the next (k+1) slice by building new 2D differential systems in slice level planes. The algorithm stops when all slices have been processed or when the object surface has been entirely described. This method allows to find automatically all the components of a complex object in which branching problems may occur and to determine how they are linked together. This possibility is one of the major advantages of the method because surface reconstruction from a set of contours is a critical step for complex structures. Classically, interpolation between contours is performed by triangulation techniques or by creating intermediate contours with dynamic elastic interpolation. But these methods need sometimes interaction with the user. In our method the surface modelling is performed in the segmentation step. This algorithm has been tested on MRI images for stereotaxy before stimulation needle introduction or brain tumour puncture [118]–[129].

#### The remarkable Gaussian line

**Figure 10:**
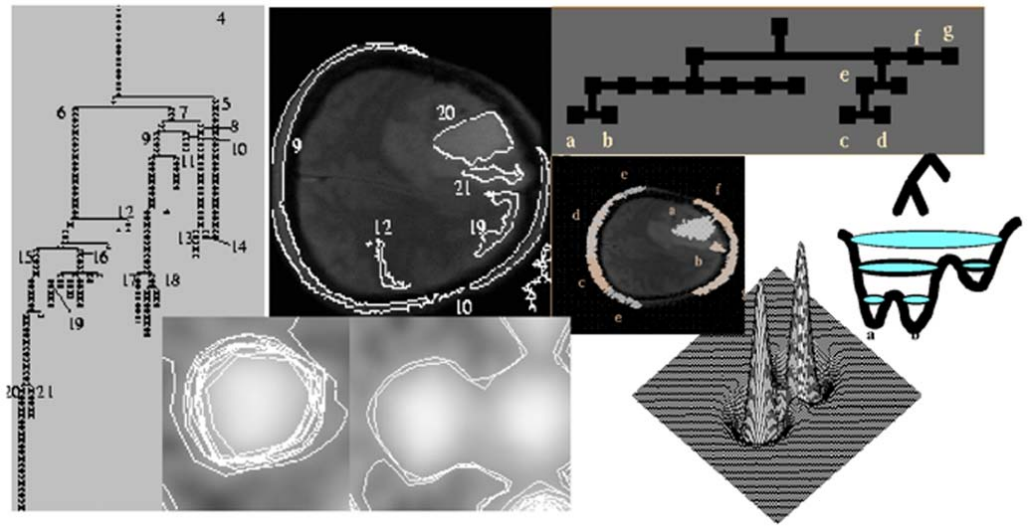
Discrete segmentation and continuous contouring. Left: confinement tree [111]. Top-middle: level sets of the confinement tree in a brain tumour NMR slice [111]. Top-right: watershed tree [103]. Middle-left: level sets of the watershed tree [110]. Middle-right: watershed tree and landscape with different water levels. Bottom-left: on the left (respectively right) successful (respectively failed) contour of the remarkable Gaussian line in case of one (respectively two) isolated (respectively close) grey level peak(s) [41]. Bottom-right: 3D image of two close peaks [41]

Homogeneity is not always a stable characteristic of an anatomical structure. So we present now a differential system performing H(g) = 0, where H is an operator similar to the Laplacian or Marr-Hildreth detectors. Let us define the remarkable Gaussian line of a peak as the set of points where the mean Gaussian curvature of the peak vanishes (Figure 10). Its equation writes [41]:
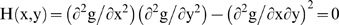



If H' = |H|, let consider the mixed potential Hamiltonian system [42]–[44] obtained as follows:
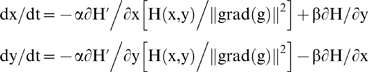



We consider in Figure 10 bottom right the new grey function H(x,y) instead of the function g(x,y) at each pixel (x,y) and we display bottom left the mixed potential Hamiltonian differential system above of which the characteristic line is a limit cycle, called the Hamiltonian contour. Its first term is of steepest descent dissipative nature and along the flow, the trajectories converge to the zeros of H'(x,y). On the set of the zeros of H'(x,y), the second Hamiltonian term of the differential system which is of conservative type, becomes preponderant. Parameters α and β can be used to tune the speed of convergence of the differential system to the limit cycle. The usual Runge-Kutta-Gear discretization scheme yields ultimately for the differential system an algorithm which is quite easy to implement. On each pixel (boundary effects are neglected), the function H(i,j) reads:
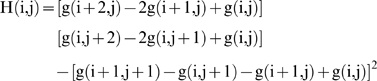



An important property of the remarkable Gaussian line is that in the case of a Gaussian peak, it contours the projection of a volume equal to 2/3 of the total volume of the peak. This property remains available with a good approximation in case of moderate kurtosis and skewness of the peak. An advantage of this technique is that we do not perform a direct segmentation of the grey level. Thus the segmentation is much finer than the corresponding one performed by the watershed lines method or by its variant with markers [103]. We only segment the upper part of the peak and then we multiply by 3/2 the activity integrated inside the remarkable line. This approach is interesting because the lower part of the peak is often noisy. The method seems particularly efficient when the peaks are well separated. If they are close (see Figure 10 bottom right), then we need to tune the parameters α and β and to start the trajectories inside the peaks. For finding a contour line inside, we can:

1. calculate the total variation V(h) = ∫_C(h)_||∇g(x)||dx of the gradient norm||−∇g|| along a contour line C(h) of level h
2. both decrease and increase h towards two limits h_1_<h and h_2_> h in order to find an intermediary value of V(h) greater than the two values V(h_1_) and V(h_2_) calculated at the extremities h_1_ and h_2_. Then C(h_1_) and C(h_2_) constitute an annulus whose intersection with the remarkable line is not empty
3. choose the initial condition on C(h_2_) for starting the simulation of the differential system.

Eventually, we can notice that the remarkable Gaussian lines can serve for matching images or objects of interest, for example, in the case of comparing images to a reference coming from an atlas. They constitute a feature in general more robust than parabolic or saddle singularities sensitive to perturbations causing local skewness of the grey peaks.

#### Watershed contouring

The watershed line is a concept firstly defined by geographers in order to characterize the main features of a landscape: a drop of rain that reaches the ground will flow down to a sea or an ocean. In the case of France, the watershed line splits the country in two parts, the Atlantic zone and the Mediterranean zone. Those zones are called ‘catchment basins’, and the oceans are the minima of them, i.e. the attraction basins of the gradient operator which corresponds to the gravitational dynamics of the drop on the steepest gradient lines of the relief surface. They define a partition of this relief, and the boundaries of catchment basins define on the pixels plane the watershed lines [105]–[109]. These lines are confounded in regular cases with the crest lines surrounding the catchment basin. It is easy to understand the interest of this concept in image processing: grey level images can be considered as relief structures, and the watershed lines are a good way to separate light (low grey level) zones from dark (high grey level) ones. It is particularly interesting to determine the watershed lines of the symmetrical reverse landscape obtained by considering the new grey level 1-g, where g is the initial normalized grey level obtained after the contrasting step and after fixing the maximum of g as a normalized value equal to 1. The watershed lines verify variational principles: i) when progressively fulfilling with water a catchment basin, its inner area passes through a series of inflexion points corresponding to the successive saddle points reached by the water. Each inflexion point corresponds to a local maximum of the second derivative of the inner area; ii) for a given inner area, the watershed lines are those containing the maximum of water. The watershed line is computed on a discrete image, by immersion simulation, locating it on the meeting points of several catchment basins (Figure 10). First discrete algorithms of watershed lines computed by immersion simulation were proposed in [105]–[109] with a discrete operator. In [103], [110], the watershed line is computed on the reverse image, in order to have one and only one local maximum of the original image into each catchment basin of the reverse image. The resulting labelling (still not a partition) is done on the original image. We used the Vincent-Soille algorithm [105] on discrete images with a linear complexity (about 7,25 n, where n denotes the number of pixels in the image). It can be used also in 3 dimensions.

#### Reaction-diffusion contrasting

Several methods of image contrasting by using differential linear or non-linear operators have been proposed [92]–[99]. These methods can be parallelized as for the neural networks and we will show in the following that there exists a deep relationship between the discrete neural network approach and the continuous differential operator approach.

1. **The Catté-Lions-Morel-Coll non-linear diffusion operator.** It is well known that the solution of the heat differential operator: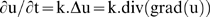 is the Gaussian kernel, with variance equal to σ^2^ = 2 kt, by choosing as initial conditions u(.,0) the grey level. This property has suggested [94] the use of another differential non-linear diffusion operator:
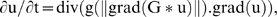
where G is a Gaussian kernel and g is a non-negative non-increasing function on R_+_ verifying g(0) = 1 and g tends to 0 at infinity; in practice, we can choose for g a set function, whose value is 1 on the interval [0,S] and 0 on]S, +∞[: there is diffusion if and only if ||grad(G_*_u)||≤S and, after a certain transient, it remains a gradient only on the boundary of sufficiently discriminable objects. For example, Figure 11 presents images after some hundreds of iterations, showing the gradient on the boundary of brain structures. The end of the procedure as for the heat operator (Figure 11 left (a)) shows that diffusion wins, giving a constant grey level at the asymptotic state. In order to improve the method of getting the contrasted image at the asymptotic state of the simulation, we must add a reaction term in order to obtain the final expected image as the attractor of a differential reaction-diffusion operator, like for the iterative discrete neural network as in Figure 11 left (c).

**Figure 11:**
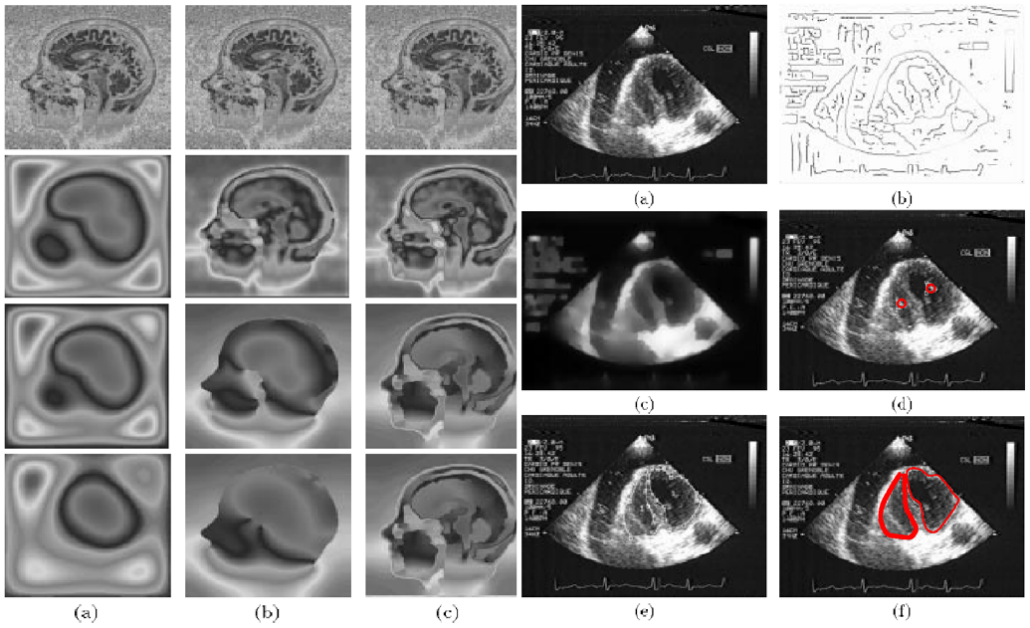
Continuous contrasting operators. Left: comparison between heat diffusion (a), Catté-Lions (b) [94] and non-isotropic (c) [93], [95], [96] contrasting operators. Right: non-isotropic contrasting operator and snakes contouring of the cardiac ventricular cavities with initial image (a), Canny-Deriche treatment [146] (b), non-isotropic processing (c), snakes splines contouring (d–f)
2. **The non-isotropic reaction-diffusion operator.** By searching a continuous operator having as discrete finite elements scheme a deterministic neural network system similar to that presented in Section 2, it has been proposed [93], [95], [96] with direct reference to the discrete neural network approach [48], [49], [52] a new reaction-diffusion operator. Let us recall the deterministic neural network with threshold 0 defined by:
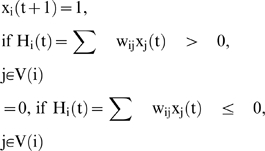
where V(i) is a neighbourhood of i. If we suppose the neural network to be 2D and infinite, lets us denote by (i_1_, i_2_) the position of the neuron i, where i ∈ Z^2^; if w_ij_ are symmetrical and translation invariant with finite range R, where R is the radius of the neighbourhood V(0) of 0, there exists T defined on [−1,1]^2^ and valued in [−1,1] such as: 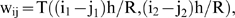
where h is a strictly positive real number, T has as mean value m, 
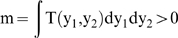
and variance 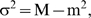
wherein 
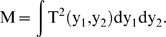
Let us denote now by f a continuous regularized version of the Heaviside function (like the arc-tan) and let us take 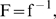


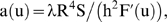


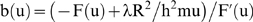
then the reaction-diffusion operator defined by: 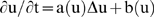
has a natural discretization corresponding to the neural network above, by identifying x_i_(t) and u(ih,t) and by remarking that the neural network system has the same asymptotic behaviour as the differential system: 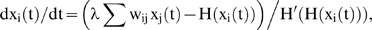
when λ is sufficiently large. In [93], it is shown that, for adapted values of R, homogeneous in grey, 1D objects can be enhanced in a heterogeneous environment, in the same way as for a neural network system. In [96] and in Figure 11 (right), the same proof is given for 2D objects like the internal cavities of the heart, where a snakes splines procedure is used after contrasting.
3. **Proposal for a new image reaction-diffusion-chemotaxis operator.** In order to have, like for the previous operator, the final treated image as asymptotic of a differential operator, we propose to consider the grey level u as a chemotactic substrate concentration consumed by animals whose concentration will be denoted by v [84], [85], [144]. The principle of this method consists in locating initially a uniform concentration v(0) of animals on the initial grey level image u(0) or on its boundary: the substrate u can diffuse with a term εΔu and is consumed with a saturation rate equal to: −Kuv/(u+k); the animal concentration v can diffuse attracted by the substrate with the term DΔv, is submitted to a drift in the direction of substrate peaks with the chemotactic term - χdiv(vgradu) and increases (because of the reproduction) with the term K'uv(u+k'). Let us remark that the two first terms ruling the animal motion can be replaced, if we do not want to introduce a drift term, by an attraction-diffusion term like: 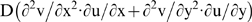 The corresponding differential partial derivative operator is then given by: 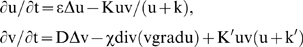 or by the following PDE: 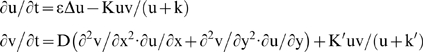
In the two cases above, the asymptote of u is 0 and the asymptotes of v give the “treated image”. The corresponding image processing leads to a contrast enhancement before segmentation: in Figure 7, we can see the initial image on the bottom left and the contrasted one on the bottom right. The contours have been then easily obtained by applying a snakes splines procedure [71], [72]. If we are adding to the second equation of the differential system a Dupin term like Kv/Δu, we will encourage animals to follow Dupin lines, i.e. inflexion curves, which is very suitable for a grey anticlines segmentation (for example in vessels segmentation).

### Conclusion

The neuro-mimetic lateral inhibition mechanism and the set valued snakes-like flows allow the generation of various image processing methods (essentially contrast enhancement and contouring). We have given numerous applications of this methodological approach in image processing essentially dedicated to medical imaging and surgical robotics. Further both theoretical and numerical studies have to be completed, in order to show the utility of these new tools in morphogenesis modelling, allowing to generate artificial objects of biological and/or medical interest (like cells, tissues, organs) by using the same operators as for recognizing them in a real image. We conjecture that the spatial information about anatomic organs obtained from the biomimetic image-processing methods, has to do with the morphogens localization, which results from the morphogenetic processes creating these organs combining robust genetic regulatory networks [145], [146] ruling their metabolic reactions and cell proliferation, with classical diffusion [147] of morphogens inside their tissues. In particular, the main patterns observed during the embryonic formation can be found in the biomimetic processing of the images by the final adult organ.
