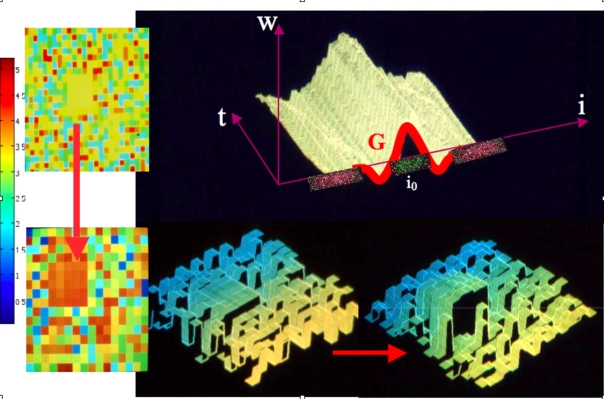# Correction: Understanding Physiological and Degenerative Natural Vision Mechanisms to Define Contrast and Contour Operators

**DOI:** 10.1371/annotation/c22f33ac-613d-4412-b0e6-db9a1cb59143

**Published:** 2009-07-31

**Authors:** Jacques Demongeot, Yannick Fouquet, Muhammad Tayyab, Nicolas Vuillerme

The published Figures 1 and 5 contain errors. Please see the corrected figures here:

Figure 1, 

**Figure:**
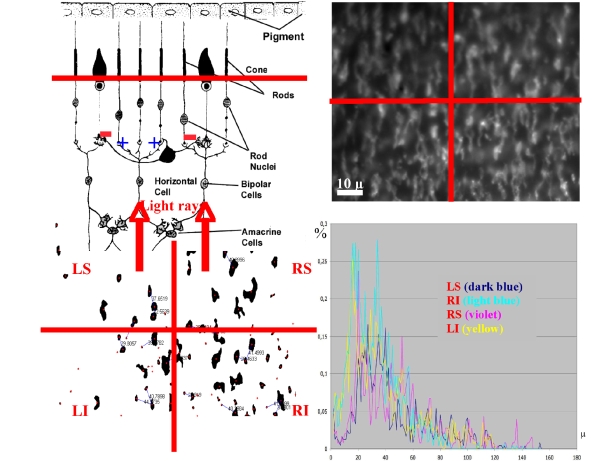


Figure 5, 

**Figure:**